# Is functional brain connectivity atypical in autism? A systematic review of EEG and MEG studies

**DOI:** 10.1371/journal.pone.0175870

**Published:** 2017-05-03

**Authors:** Christian O’Reilly, John D. Lewis, Mayada Elsabbagh

**Affiliations:** 1 Douglas Mental Health University Institute, 6875 Boulevard Lasalle, Verdun, Canada; 2 Department of Psychiatry, McGill University, 1033 Pine Avenue West, Montreal, QC, Canada; 3 McGill Center for Integrative Neuroscience, Montreal Neurological Institute, McGill University, 3801 University Street, Montréal, QC, Canada; Istituto Italiano di Tecnologia, ITALY

## Abstract

**Background:**

Although it is well recognized that autism is associated with altered patterns of over- and under-connectivity, specifics are still a matter of debate. Little has been done so far to synthesize available literature using whole-brain electroencephalography (EEG) and magnetoencephalography (MEG) recordings.

**Objectives:**

1) To systematically review the literature on EEG/MEG functional and effective connectivity in autism spectrum disorder (ASD), 2) to synthesize and critically appraise findings related with the hypothesis that ASD is characterized by long-range underconnectivity and local overconnectivity, and 3) to provide, based on the literature, an analysis of tentative factors that are likely to mediate association between ASD and atypical connectivity (e.g., development, topography, lateralization).

**Methods:**

Literature reviews were done using PubMed and PsychInfo databases. Abstracts were screened, and only relevant articles were analyzed based on the objectives of this paper. Special attention was paid to the methodological characteristics that could have created variability in outcomes reported between studies.

**Results:**

Our synthesis provides relatively strong support for long-range underconnectivity in ASD, whereas the status of local connectivity remains unclear. This observation was also mirrored by a similar relationship with lower frequencies being often associated with underconnectivity and higher frequencies being associated with both under- and over-connectivity. Putting together these observations, we propose that ASD is characterized by a general trend toward an under-expression of lower-band wide-spread integrative processes compensated by more focal, higher-frequency, locally specialized, and segregated processes. Further investigation is, however, needed to corroborate the conclusion and its generalizability across different tasks. Of note, abnormal lateralization in ASD, specifically an elevated left-over-right EEG and MEG functional connectivity ratio, has been also reported consistently across studies.

**Conclusions:**

The large variability in study samples and methodology makes a systematic quantitative analysis (i.e. meta-analysis) of this body of research impossible. Nevertheless, a general trend supporting the hypothesis of long-range functional underconnectivity can be observed. Further research is necessary to more confidently determine the status of the hypothesis of short-range overconnectivity. Frequency-band specific patterns and their relationships with known symptoms of autism also need to be further clarified.

## Introduction

It is well recognized that autism and autism spectrum disorder (ASD)–hereafter used interchangeably–is associated with altered patterns of connectivity, compared to neurotypical (NT) controls. Increased interest in connectivity reflects a shift from understanding the biological basis of autism as focal brain abnormalities affecting specific systems towards an overall pattern of brain reorganization. Moreover, new evidence on early development of white matter tracks suggests that connectivity could be among the earliest markers of autism, with initial signs emerging within the first year of life [1–3].

Although the autistic brain was initially hypothesized as exhibiting a pattern of overall underconnectivity [4,5] or by long-range underconnectivity and local overconnectivity [6], a more subtle mixture of hypo- and hyper-connectivity is now emphasized [7]. Often, findings remain unreplicated and conclusions divergent regarding the nature of altered connectivity in autism. Several reasons may explain the differences in findings and conclusions including conceptual (e.g., definitions, theoretical models), methodological (e.g., measurement modalities and paradigms, participant characteristics), or analytical (e.g., quality control and processing pipelines).

Previous literature reviews have partially addressed questions about connectivity in autism. These reviews have predominantly focused on structural connectivity using diffusion imaging [8–10] and correlated activity using functional magnetic resonance imaging (fMRI) [10–12]. In contrast, other aspects of connectivity including functional and effective connectivity of electrophysiological activity reported in electroencephalography (EEG) and magnetoencephalography (MEG) have not been the focus of systematic synthesis (however, see [13] for a narrative synthesis of coherence in EEG resting state). Yet, such syntheses are paramount in getting a clear view of the relationship between brain connectivity and autism considering that 1) different recording modalities can provide contrasting points of view on mechanisms altering brain connectivity (e.g., synaptic functions, degree of myelination, inhibitory/excitatory balance, network properties) and 2) their results are not necessarily in good agreement [14]. Because of their high temporal resolution and their direct relationship with neuronal activity (as opposed to a proxy such as hemodynamic), EEG/MEG connectivity analyses can provide valuable information about dynamic activation and deactivation of functional networks. Further, these can be observed for different oscillatory frequencies depending on the role of each network in integration versus segregation of information, on top-down versus bottom-up propagation of signals, and the tasks or functions they support. Synthesizing evidence about altered functional network connectivity in autism is essential for establishing a coherent theoretical account of the pathophysiology of the condition. Thus, our goal is to fill knowledge gaps by comprehensively reviewing literature on EEG/MEG functional and effective brain connectivity in autism, with a focus on factors influencing over versus under connectivity. We begin with an overview of relevant connectivity concepts and measurement approaches.

### Connectivity: A multi-faceted concept

Brain connectivity is a broad, multi-faceted concept [15]. In human neuroscience, connectivity can refer to physical interconnection of brain regions through bundles of axons (*structural*/*anatomical connectivity)*, to statistical dependencies (e.g., correlation, coherence, consistency in phase-lag) between time series of cerebral activity in different brain regions (*functional connectivity*), or to causal interactions between brain regions (*directed*/*effective connectivity*) [16]. Structural/anatomical connectivity is generally assessed with deterministic or probabilistic tractography of diffusion weighted images recorded using magnetic resonance imaging (MRI) scanners. The two other types (i.e., functional and effective) are assessed mainly using electromagnetic (e.g., electroencephalography (EEG), magnetoencephalography (MEG), local field potentials (LFP), spike trains), hemodynamic (e.g., functional MRI (fMRI) or near infra-red spectroscopy (NIRS)) and, to some extent, nuclear recordings (e.g., positron emission tomography (PET), single-photon emission computed tomography (SPECT)).

Although the term *connectivity* is often used interchangeably in the literature to denote any or all of these variants, different measures can show surprisingly little agreement [14,17]. Aside from measurement issues and biases, a few reasons may explain the discrepancy among measures. One of these reasons is the complexity of the relationship between structural and functional connectivity. For example, neural networks have an intricate structure of excitatory and inhibitory neurons forming local microcircuits (i.e., “a minimal number of interacting neurons that can collectively produce a functional output” [18]), which synaptic connectivity can either amplify or attenuate measures of functional connectivity. How this micro-connectivity impacts on macroscopic structural and functional connectivity is unclear. Functional connectivity can also be modulated by factors independent from structural connectivity (e.g., synaptic depression, properties of sensory afferent signals) and can appear through indirect paths, not structurally connected by a direct track but functionally coordinated by an intermediate structure (e.g., cortico-thalamo-cortical pathways) not considered by direct structural connectivity.

Similarly, different modalities of functional connectivity show complex interdependencies. For example, the relationship between electrical and hemodynamic activity has been shown to depend on frequency and spatial scale, with particular EEG rhythms generating region-dependent variations in blood oxygen levels and glucose metabolism [19,20]. Cross-frequency dependencies can even be observed between recording modalities. For example, slow hemodynamic rhythms are known to be correlated with the amplitude of fast gamma-band EEG/MEG activity [21,22].

Furthermore, it is worth remembering that functional connectivity is generally based on similarity of signals observed between pair of regions and, in most cases, no control of the potential contribution of a third sources is made. This situation may be improved by multivariate approaches [23], but it seems unlikely to be completely controlled, particularly for cortical activity initiated by subcortical structures (e.g., thalamo-cortical pathways) since such third sources are generally hidden (i.e., not measured) and are therefore difficult to estimate and mitigate [24]. The situation is much different when assessing structural connectivity by measuring the area of a fiber bundle section linking two regions. In the latter case, there is no potential hidden third source confound. Therefore, different forms of connectivity need to be treated as different, yet related, constructs. Integrating knowledge from EEG/MEG with other functional (e.g., fMRI, PET) or structural (e.g., tractography) modalities is expected to be a fruitful avenue because of possible complementarity between modalities, but it must be performed very cautiously in view of many potential pitfalls.

### Connectivity hypotheses in ASD

The large amount of literature on connectivity in autism yields multiple distinct hypotheses about the nature of over- and/or under-connectivity. We considered three classes of inter-related hypotheses regarding connection length, topological specificity, and developmental effects.

#### Over- and under-connectivity in relation with connection length

A popular hypothesis considers autism to be characterized by long-range underconnectivity potentially combined with local overconnectivity [6,25–27]. This hypothesis is predominantly supported by structural and functional MRI and post-mortem immunocytochemistry investigations. For the structural part, this pattern can be explained by ASD-related abnormalities at the cellular level:

- long-range structural underconnectivity can result from a degradation of fiber bundles [1,28–40], with many studies reporting decreased fractional anisotropy and/or higher mean diffusivity in ASD for the superior longitudinal fasciculus, occipitofrontal fasciculus, uncinate fasciculus, inferior longitudinal fasciculus, cingulum, and corpus callosum [41];
- local structural overconnectivity can result from a decrease of apoptosis, axonal pruning, and dendritic degradation, and from an increase of neurogenesis [42].

However, local connectivity may often be obfuscated by long-distance connections (e.g., in presence of long-range crossing fibers in diffusion imaging). In such cases, long-range underconnectivity makes local connectivity more clearly observable without meaning that there is a true increase in local connectivity (i.e., there is an augmentation of the relative number of local connections because of a loss of long-distance connections, but there is no augmentation in their absolute number).

For the functional part, many fMRI studies reported long-range underconnectivity in ASD, whereas local overconnectivity has been reported less consistently [26,43,44]. In a recent review of the literature, 26 out of 33 fMRI studies were shown to report reduction or loss of–sometime local but most often long-range–connectivity in ASD [41]. The prefrontal cortex and the posterior cingulate cortex were most often shown to exhibit long-range underconnectivity. Other regions (e.g., precuneus, anterior cingulate cortex, superior temporal gyrus, posterior superior temporal sulcus, anterior insula, parietal lobule) showed primarily long-range underconnectivity, but were also associated in some studies with long-range overconnectivity.

Various experimental observations have been proposed as potential correlates or causes for local functional overconnectivity, such as smaller but more numerous cortical neurons and mini-columns, which might indicate a bias toward local processing [45]. It may also be caused by a higher excitatory/inhibitory ratio favoring local interactions [46,47], for example through a deficient GABAergic signaling [48]. Appropriate GABAergic activity is also important for normal operation of local circuitry such as appropriate functional segregation of mini-column through lateral inhibition provided by GABAergic basket cells [49]. It is also involved in generation of gamma-band activity through parvalbumin-expressing fast-spiking interneurons [50]. Activity in the gamma-band is associated mostly with local computation [51,52], it is involved in many processes (e.g., perceptual binding and selective attention [53]) showing alteration in ASD, and abnormalities in this frequency band have been reported consistently enough to be proposed as being a marker of ASD [54].

Finally, related to the hypothesis concerning long-range versus short-range connectivity, the operational definition in the reviewed literature remains elusive. This is particularly problematic in EEG/MEG, since these modalities do not have a good spatial accuracy and comparison of nearby pairs of sensors (i.e., local connections) is confounded with volume conduction (see the discussion for more on this topic). One possible definition–and the one we use in this review–for EEG/MEG long-range connectivity is that it reflects inter-lobar or inter-hemispheric connections. However, we also interpret evidence showing a graded response of connectivity with respect to distance, for example, when the connectivity is correlated with the inter-sensor distance.

#### Topological specificity

Evidence for altered connectivity in various brain regions comes from investigations of brain structure (MRI, diffusion imaging, post-mortem immunocytochemistry, etc.). ASD-related abnormalities have been documented in the frontal lobe [25,55,56], including abnormal organization of neurons and microglial cells [57]. Evidence from fMRI supports a pattern of underconnectivity from frontal to other brain regions [58]; i.e., lower frontal to parietal connectivity [59] and reduced antero-posterior connectivity [58,60]. However, these studies were conducted in adults and may therefore be characterizing a cascade effect that appears over development. For example, underconnectivity in 24-month-olds with ASD have been shown to be predominantly in occipital regions, with important abnormalities in temporal lobes, but almost no abnormalities in frontal areas [61].

Abnormal connectivity between the occipital lobe and the other regions is also often reported; an observation that might be related with structural and functional abnormalities in processing of visual input in ASD [62–66]. In a large database of resting-state fMRI recordings, underconnectivity was also found in all lobes, but particularly for the temporal, whereas overconnectivity was mainly affecting connectivity with subcortical structures, particularly for connections linking the thalamus and the globus pallidus to the primary parietal sensorimotor regions [67].

Contrary to hypotheses about deficiency in specific regions, an alternative hypothesis suggests an overall non-topographically-specific alteration of brain connectivity in ASD. Support for this hypothesis is based on evidence of a more randomly connected brain in ASD [68], which results in a cross-interaction between the degree of connectivity of brain regions and the diagnosis of ASD; i.e., ASD shows an increase (respectively, a decrease) in connectivity for pairs of regions which display low (respectively, high) connectivity in controls [69].

#### Developmental hypotheses

The typical developmental course of connectivity remains poorly understood but its determinants (e.g., pruning, myelination) and its indexes (e.g., fractional anisotropy, magnetization transfer ratio) appear to follow an inverted U-shape trajectory during maturation, which progress from posterior to anterior regions and from primary to association cortical areas. These maturational patterns appear to differ in autism from very early in development [1,2]. Diffusion imaging shows early elevated fractional anisotropy (1.8–3.3 year-old range in [70]; 1.5–5.8 year-old range in [71]; elevated at 6 month-old followed by reduction to below controls at 24 month-old in [1]) followed by a reduction below neurotypical values later in life (7–33 year-old in [28]; 14.6 ± 3.4 year-old in [72]). This observation suggests a possible age-related inversion of trends in measured connectivity (i.e., from initial overconnectivity to later underconnectivity) potentially due to slight differences in developmental trajectories. Interesting relationships might be hypothesized linking this potential initial overconnectivity with the early overgrowth of the brain in ASD [55,73] and the early maturation of white matter tracts previously reported in toddlers and young children with ASD [1,70,71,74]. Because early stages of typical development involve initial structural overconnectivity followed by a pruning of connections in the maturing brain [75], differences in maturation rates can be expected to modulate the degree of connectivity. For example, a delay in the onset of axonal remodeling could drive brain overgrowth, yielding an abnormally large brain and therefore abnormal long-distance connections. Such connections are more likely to be pruned given that this process is driven by competition for neurotrophins [76], and the increased conduction delays and cellular costs associated with longer fibers puts them at a disadvantage in this competition. Thus, a delay in the onset of axonal remodeling could drive brain overgrowth and thereby favor short-distance connections over long-distance connections via typical developmental mechanisms [77,78].

Differences in developmental trajectories of brain connectivity observed with EEG/MEG might also be exacerbated if excitatory and inhibitory neurons mature at different speeds (e.g., a slower rate of reduction of intra-cellular chloride ionic concentration during development would result in delayed transition of GABAergic neurons from excitatory to inhibitory [48]). Such an altered development would shift the normal evolution of the excitatory/inhibitory ratio (already known to be abnormal in ASD [47]). In turn, it is likely that this imbalance would influence the degree of functional connectivity in cortical pyramidal neurons, the cell population generally considered as the main source for observable EEG/MEG activity. Similarly, differences in maturation of feedforward versus feedback pathways might be responsible for some transient developmental aberration in overall connectivity. Since feedback connections develop later than feedforward [79] and ASD is a developmental disorder, feedback projections could be preferentially altered.

Regarding which brain region might show the largest developmental differences, fMRI studies have reported the default mode network as being particularly vulnerable to ASD [80,81]. The associated brain regions would show a slower maturation than in neurotypical controls. Among the structures involved in this network, the impact of slower maturation is likely to be more prominent for the frontal lobe since it is one of the last structures to mature in the brain, a process that continues up to the mid-twenties [82].

### Scope of the review

Taken together, these different hypotheses demonstrate the complexity of the impact of ASD on connectivity, which is most probably characterized by both generalized atypicality (e.g., long-range underconnectivity, increased randomness in connection patterns) and more localized abnormalities (i.e., specific deficits in some nuclei or brain regions) that develops during the first years of life.

Although the current body of evidence related to connectivity comes mostly from fMRI [10–12] or from structural approaches using diffusion imaging [8–10], there has been an increase in complementary methods. Specifically, EEG/MEG functional and effective connectivity can reveal qualitatively different results which are important to our understanding of how the brain wiring is altered in autism. Evidence synthesis on that topic is currently missing. Therefore, in this paper, we comprehensively review the literature on EEG/MEG functional and effective brain connectivity in autism, against the different hypotheses described above, particularly focusing on factors influencing overconnectivity versus underconnectivity. Although it is currently unclear if local connectivity in EEG/MEG should be assessed by comparing the activity between pairs/groups of sensors or alternatively using single point measurements across a whole group of sensors (e.g., through spectral power), this review is limited to studies measuring connectivity using the former approach. Complementary review of other novel methods to characterize brain activity in ASD such as quantitative EEG can be consulted elsewhere (e.g., see [83,84]).

## Methods

Our review focuses on original research assessing functional and effective connectivity as measured by EEG or MEG. Searches on PubMed and PsychInfo executed on March 6^th^ 2016 using the research string “(EEG OR MEG OR electroencephalo* OR magnetoencephalo*) AND connectivity AND autis*” returned 102 and 67 hits respectively. Only journal and conference papers published in English were reviewed.

Studies were retained if they presented original experimental findings on participants with an ASD phenotype, including individuals diagnosed at any age as well as studies comparing low versus high-risk infants defined by the presence or absence of an older sibling diagnosed with ASD. One study correlating ASD traits in a neurotypical population with differences in connectivity features was also included.

We excluded articles discussing functional or effective brain connectivity without reporting new experimental results on this topic. We also excluded articles reviewing neurofeedback approaches using connectivity measurements, methodological articles proposing new approaches for assessing brain connectivity, and studies inferring on connectivity only through indirect measurements, e.g., EEG/MEG complexity, ERP timing, spectral power, or local inter-trial synchronization.

Appraisal of methodological quality of included studies was conducted by one of the authors (COR). This led to exclusion of one study due to probable methodological issues related to multiple comparisons, confounders, and mismatching statistics [85], and of a second study because of a too small sample (i.e., two subjects per group [86]). A third study [87] was retained but interpreted with caution because of inappropriate control of multiple statistical comparisons. In that study, the authors addressed the problem of multiple comparisons by computing, for each node of their graphs, an average t-value from 48 leave-one-subject-out t-tests. However, if standard statistical hypotheses are valid (normality, absence of outliers, etc.), this “bootstrapped” evaluation of the mean t-value will converge toward the t-value for the whole sample (i.e., the uncorrected t-value). However, t-tests are still being computed at every node resulting in N independent statistical tests with an alpha-threshold at 0.05, which will provide up to 5% of false positives under the null hypothesis. The “leave-one-out” approach does nothing to correct for these multiple comparisons.

**Fig 1:**
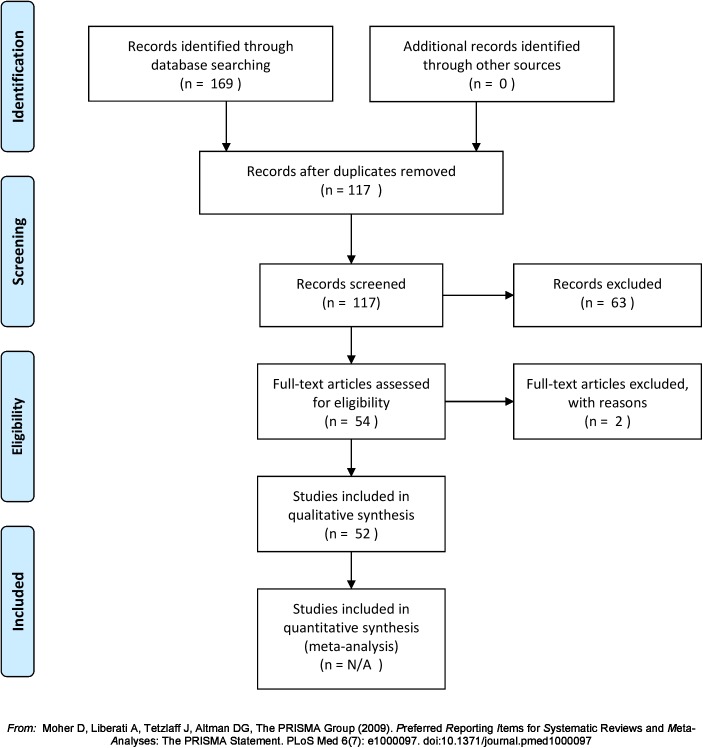
PRISMA (Preferred Reporting Items for Systematic Reviews and Meta-Analyses) flowchart describing the paper selection process.

A total of 52 papers were retained, 31 using EEG [88–118] and 21 using MEG [87,119–138]. The selection process is depicted in Fig 1. For each study, one of the authors (COR) extracted methodological dimensions that potentially impacted results and conclusions, such as:

- connectivity metrics;
- recording parameters (sampling frequency, reference electrode, electrode grid density);
- experimental paradigms (e.g., resting-state, event-related experiments, sleep studies);
- sample characteristics (age, gender, IQ, diagnostic confirmation method, sample size).

The wide variability in study characteristics along these methodological dimensions precluded a meta-analysis to address the main objectives of the review. Instead, we synthesized and critically appraised findings of the over-arching hypothesis that ASD is characterized by long-range underconnectivity and local overconnectivity (as a valid generalization of a more complex phenomenon), and systematically reported results related to factors that can impact this hypothesis (exceptions to the generalization; development, topography, lateralization).

## Results

### Synthesis per methodological dimensions

We first synthesized included studies across several methodological dimensions that may account for some inconsistencies in including connectivity metrics, recording parameters, experimental paradigm, and sample characteristics. Outcome of this analysis can be found as supplementary documents S2 File along with tables (see S1 and S2 Tables) summarizing the information extracted for included studies (split by EEG vs. MEG).

### ASD is characterized by long-range underconnectivity; Local overconnectivity remains uncertain

As reviewed in the introduction, a prominent hypothesis is that autism is characterized by local overconnectivity and long-range underconnectivity [25,26]. EEG/MEG studies provide partial support for this hypothesis with some studies showing both phenomena in ASD population [90,114]. This distribution of under/overconnectivity is also correlated with the presence of autistic traits in the neurotypical population [89].

Overall, the case for long-range underconnectivity is well supported. This is particularly clear for the case of inter-hemispheric connections, for which a decrease in EEG/MEG functional connectivity has been reported in many studies [93,94,96,100,107,114,134]. A smaller number of studies report mixed long-range overconnectivity and underconnectivity [97] or increased long-range connectivity in infants [113] and adolescents [116,117], in adult rapid-eye movement (REM) sleep [108], and during a picture-naming task in adults [120]. Also, some indication for long-range overconnectivity has been found during slow wave sleep [92], although the effect was marginal (p-values = [0.1, 0.05]) when corrected for multiple comparisons. In sum, while ASD is characterized by a general long-range underconnectivity, it is likely that this pattern is modulated by task requirements or developmental processes.

Local overconnectivity in ASD is less robustly established. A few studies report local overconnectivity [89,90,114,117,139], while others found local underconnectivity in ASD [96,97,125,130,140] or a mix of both patterns [112]. Similarly, indirect support for local overconnectivity is provided by reports of an enhanced local synchronization [141], which can be associated with higher local functional connectivity (i.e., functional connectivity is correlational in nature and assesses how the activity between two regions is similar, which often means synchronized). There are, however, many theoretical and practical issues related to the definition and the reliable measurement of local connectivity in EEG/MEG (see the Discussion section) which make it difficult to reach definitive conclusions.

**Fig 2:**
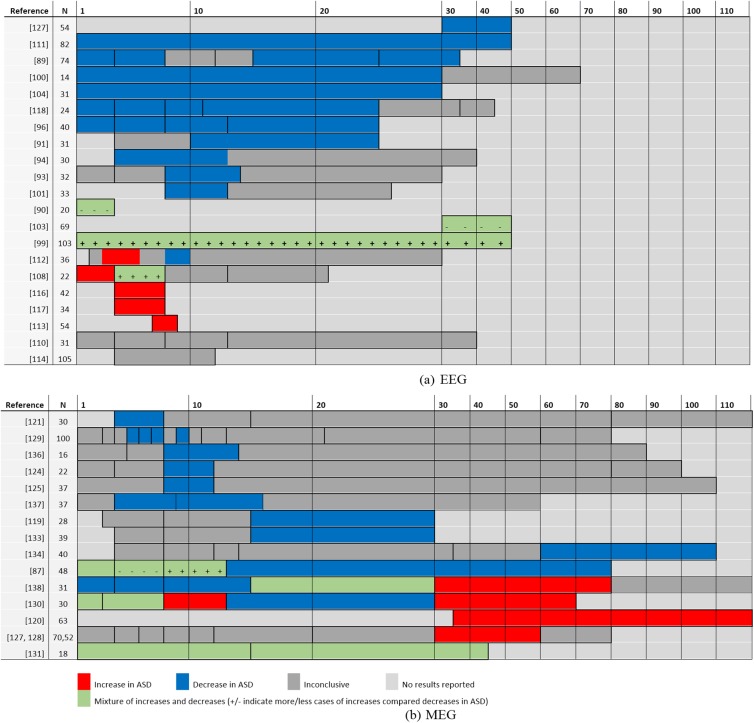
Summary of band-specific increases versus decreases in EEG/MEG functional connectivity in ASD (compared to NT controls). The “N” column list the total size of the sample (i.e., sum of participants in all groups). Frequency is varying along the x-axis, from 1 Hz to 120 Hz.

The hypothesized pattern of long-range underconnectivity and short-range overconnectivity is paralleled by the general trends observed as a function of frequency (see Fig 2). Overall, evidence suggests underconnectivity in ASD at lower frequencies (delta to beta bands) with potential overconnectivity in higher bands. This tendency is particularly well illustrated in resting-state by the work of Ye and collaborators [138]. These general trends seem rather robust despite the substantial methodological variability across studies, e.g., paradigms, samples, and connectivity measures (S1 and S2 Tables). This pattern of results is different from reported EEG power abnormalities in autism, which show a U-shape curve with increased low-frequency (delta, theta) and high-frequency (beta, gamma) activity and reduced middle-ranged frequencies (alpha) in ASD [13].

General underconnectivity in lower frequency bands has been observed as a function of the number of autistic traits in a neurotypical sample [89] as well as for all age groups of ASD participants (e.g., in infants and young children [91], in adolescents [93], and in adults [94]) and for a wide range of paradigms (e.g., resting-state [96], event-related [100], and sleep [105]), suggesting that it is associated with neurodevelopmental abnormalities that are not limited to a specific brain region or state [93]. Evidence of overconnectivity in high-frequency bands is more scarce but accumulating, particularly in MEG studies (see Fig 2).

Tentative relationships can be proposed between frequency bands, the scale of connectivity, and the degree of connectivity. The modulation of over vs. under connectivity depending on the frequency band is consistent with slower oscillators involving more neurons in larger volumes [52]. Such a relationship is to be expected from “wiring economy”. Indeed, the coordination of higher frequency activity across regions requires faster communication. Therefore, for any given distance between two regions, larger (and costlier) axons are required to coordinate faster frequencies. In this context, it is more efficient to bias connectivity such that high-frequency band activity is shared locally, whereas slower frequency bands are used for long-range interaction. Thus, we expect high-frequencies to be *preferentially* associated with more localized processes and activity in lower bands to be *preferentially* associated with more wide-spread integrative processes. Indeed, integrative top-down processes (i.e., processes integrating *a priori* knowledge about the world with incoming signals from the senses to generate a percept) involving long-range connections are *often* associated with slower rhythms (delta, theta, alpha), whereas synchronization of local cortical networks through bottom-up processes (i.e., processes modifying the internal representation of the world to minimize its mismatch with information from the senses) *tends* to be associated with faster frequencies (beta, gamma) [142,143]. Such a generalization, however, will need further investigation in view of several exceptions (e.g., the existence of large-axon long-range fast-spiking interneurons synchronizing high-frequency rhythms between distant regions [144]). Further, both reports of MEG underconnectivity in high-frequency bands are associated with long-range connections [87,134], with one of these studies explicitly limiting its investigation to long-range inter-hemispheric connections [134].

### An alternative hypothesis from graph theory: A more uniform altered connectivity in ASD

EEG and MEG connectivity studies using graph analysis generally report autism to be associated with sub-optimal network properties, such as less clustering, larger characteristic path, reduced eigenvector centrality (a measure of the importance of nodes as communication hubs), and an architecture less typical of small-world networks [89–91,114,133,135,136]. Small-world networks are thought to be striking an optimal balance between integration and segregation, making them particularly efficient. This topography is present in a wide range of contexts, such as in social networks or flight networks, but also in neural networks [145]. It is characterized by each node having a relatively small number of neighbours but being able to reach any other node by only a small number of steps (i.e., although they tend to form small cliques, each node is separated from every other node by only a few levels of separation, thanks to a relatively small number of “hub” nodes providing between-clique connectivity).

Neural networks in ASD have been shown to be more resilient because of their greater homogeneity and their more uniform architecture [114]. This, in turn, results in a less optimal balance between local specialization (segregation) and global integration [145]. Findings from network analysis seems consistent with the hypothesis of a more randomly connected brain. It has been supported as supported by EEG/MEG studies showing smaller patches (more localized, less blended) of increased MEG signal complexity in controls compared to ASD [122], a much more disorganized pattern of connectivity in ASD [119], and more redundant networks in ASD (i.e., more randomness in a network increases redundancy and resilience but decreases efficiency and specialization) [114].

However, these analyses have been performed on graph topologies, which preserve very little relationship with spatial properties of brain activity. Specifically, edges of these graphs are not weighted by the length of the connections and the angles between the edges are unspecified. Therefore, the spatial arrangement of the nodes is lost and the results cannot be reconciled with the findings reviewed above on the impact of connection length on over/underconnectivity.

### Factors modulating connectivity patterns

Although the hypothesized pattern of over and under connectivity in autism is a valuable conceptual generalization, many factors may modulate connectivity at a finer scale reflecting context-dependent modulations in connectivity. Below we consider the factors that were prominent in the reviewed of the literature.

#### Lateralization

Many cognitive processes display brain lateralization, language being a classical example. Since symptoms associated with autism are associated with lateralized cognitive functions (e.g., executive functions and language), many studies have looked for and found atypicalities in lateralization of brain functions in autism [146]. In the reviewed literature, many studies reported function- or structure-dependent EEG/MEG connectivity abnormalities related to ASD. The most evident are reports of abnormal lateralization of functional connectivity, with an elevated left-over-right EEG and MEG functional connectivity ratio in ASD.

For example, studying gamma-band connectivity in relation with face processing, a leftward (instead of the normal rightward) lateralization emerging around one year of life was found [103]. The authors hypothesized that this abnormal lateralization might be related with potential differences in face recognition in autism [147,148] because it suggests a more featural-based (processing and recognition of local features in a visual scene; typical of left hemisphere) than configural-based (integration of all the parts of a stimulus in a coherent percept; typical of right hemisphere) face recognition processes [149]. Also, in one-year-old infants at high-risk for autism (HRA) that have later been diagnosed with ASD, an increase in the alpha-band connectivity predominant in the left fronto-centro-parietal region during video watching was found in one study [113], although a second study did not report any hemispheric differences between HRA infants and low-risk controls [115].

Similarly, an elevated long-range fronto-posterior functional connectivity in children with ASD was observed prominently in the left-hemisphere [116]. Such increased left-hemisphere coherence has also been observed in low-functioning ASD children when compared with high-functioning ASD children [117].

A rightward reduction of beta-band connectivity (phase lag index) between the occipital lobe and frontal, temporal, and parietal areas in a numerosity task (i.e., participants had to estimate the number of dots either distributed to form an animal shape or randomly positioned) was also reported in ASD [119]. The authors suggested impairment in the capacity of using a global interpretation style (Gestalt perception; right hemisphere) in ASD and potentially a shape recognition strategy relying too heavily on a local processing of visual information (left hemisphere) [150]. This hypothesis seems to be coherent with the observed decrease in long-range connectivity in ASD given that such connectivity is thought to be important for global integration of widespread brain activity. Also consistent with an elevated left-over-right functional connectivity ratio in ASD is the report of increased connectivity (Granger causality) 1) from the left inferior frontal gyrus to the left fusiform area in high-beta and low-gamma frequencies and 2) from the left superior temporal gyrus to the left occipital lobe in the beta and gamma bands during a picture naming task [120]. Another study showed reduced right hemisphere temporal-central alpha-band coherence in ASD adolescents compared to neurotypical controls [101].

In an event-related paradigm in which emotional stimuli evoke bilateral activation of the insula in neurotypical adults [151], a less bilateral pattern (i.e., more lateralized) was found in ASD with significantly lower connectivity (phase lag index) of the right (but not the left) insula with areas including the right fusiform, right inferior temporal gyrus, and superior frontal regions in ASD [133].

Using photic stimulation, a leftward predominance in connectivity increased at stimulated frequencies has been observed in ASD, with a larger group difference at higher frequencies [106,107]. Although these studies did not report such asymmetry in their resting-state recordings, Coben et al. [96] found an increase in relative theta power but a reduced absolute beta power over the right hemisphere for the autistic group during resting-state. These authors argue that such an excess in theta power might be related to abnormal brain functioning in ASD, based on similar observations made in children with attention deficit/hyperactivity disorder [152], learning disabilities [153], and mental retardation [154]. Leftward asymmetry and altered EEG power lateralization was also reported [155]. Still in resting-state, Murias et al. [112] reported theta-band overconnectivity in ASD within left hemisphere frontal and temporal cortex, whereas Barttfeld et al. [90] observed an increase in delta-band local connectivity in lateral frontal electrodes, which was particularly salient in left hemisphere.

During the REM phase of sleep, more important increases in coherence were also noted in the left hemisphere, particularly for the delta band [108]. Kikuchi et al. [127] reported the more contradictory results with an increased rightward connectivity lateralization in gamma band in a sample of 3–7 years old infants with ASD, but not on a 5–7 years old sample [128] during passive video watching. Although they found no between-group differences in laterality in that latter sample, within the ASD sample, they showed that more rightward laterality in connectivity was correlated with better performances in reading and in pattern reasoning. This associate a leftward lateralization with more severe symptoms, as shown in the other studies, and is consistent with the idea that such spatial skills typically relies more on the right hemisphere [156].

In sum, a consistent pattern of increased left-over-right EEG and MEG connectivity ratio has been observed in ASD. This pattern may well reflect specific perceptual characteristics of this population in which local components are more strongly processed (segregation) relative to global relationships between components (integration; Gestalt style). This altered processing style can be hypothesized to be a consequence of the observed drop in long-range connectivity. A lack of proper long-range connectivity may not provide an adequate substrate for the normal development of the rightward more global and integrative style of processing. Compensatory development of more leftward local-processing style may, however, still be possible if shorter-distance connectivity is not as severely affected by the disease. It is also relevant to note that an enhanced EEG power has been consistently reported in the left hemisphere across all frequencies (reviewed in [13]) which may be a confounding factors in studies not controlling for potential impact of an increase in power on the resulting increase in connectivity (e.g., as can be seen in the case of coherence computed using a common reference [157]).

#### Topography of connectivity differences in ASD

Cognitive processes that are known to be atypical in ASD are, to some extent, localized to specific brain regions. Thus, there is an interest in investigating the topography of connectivity abnormalities to further associate altered patterns of connectivity with observable phenotypes and symptoms. Among the reviewed studies, some discussed general topological differences at sensor-level, whereas others used more precise analyses of cortical sources to localize abnormalities. For example, in MEG, a reduced coherence between the frontal eye field (a region involved in voluntary eye movements) and dorsal anterior cingulate cortex in an ASD population in a saccade/anti-saccade task has been shown [124]. Abnormal underconnectivity of the fusiform face area with various other structures (left precuneus, right inferior temporal gyrus, and superior frontal regions) in tasks involving face stimuli has also been reported using cross-frequency coupling [125] and phase lag index [133].

These results are examples showing that more subtle patterns of connectivity are most certainly region- and function-dependent. Consistent with the topological hypotheses reviewed in the introduction, a large number of studies included in S1 and S2 Tables report involvement of frontal [90,93,108,112,113,121,137,138,141] or occipital regions [90,96,100,108,112,121,122,138,158], although reports of significant differences in connectivity can be found between virtually all pairs of brain regions. The variability in experimental paradigms, analyses, and sample characteristics prevents establishing clearer generalizations of these findings. It is worth noting though that frontal and occipital regions have the longest inter-hemispheric connections, which may account for the prevalence of observed underconnectivity in these regions if long-range connections are preferentially affected by ASD. Also, studies in infants are under-represented in this literature, which may cause abnormalities emerging at this age to be under-represented. Further, some regions of the brain may also be poorly covered by small-grid EEG studies, such as inferior regions of temporal lobes.

#### Development

Differences in EEG/MEG functional connectivity are emergent rather than static over development [103,115]. However, a consistent portrait of how these connectivity differences emerge is yet to be established. Righi et al. [115] reported a decrease of connectivity (coherence) in the gamma band for HRA infants, a trend that was more pronounced for the portion of HRA infants which later developed ASD, whereas Orekhova et al. [113] reported increased alpha-band connectivity (debiased weighted phase lag index) in HRA infants who later developed ASD. Relying on reported correlation between EEG alpha-band coherence and structural integrity of white matter [159], these authors relate this alpha-band hyper-connectivity in toddlers with the frequently reported alpha-band hypo-connectivity in adolescence/adulthood by highlighting the abnormal trajectory of white matter maturation in ASD: early maturation of white matter tracks in toddlers and young children [1,70,71,74] followed by slowing of white matter increase in toddlerhood [1] and later childhood [160,161] ending in predominant hypo-connectivity in adulthood [26]. Thus, patterns of connectivity differences between ASD and NT participants should not be thought of as being static over time. Although more corroboration is still needed, it might well be captured by a general early hyper-connectivity followed by a regression toward hypo-connectivity later in development.

Developmental change continues to impact connectivity patterns later in development. Using resting-state MEG recordings from 6–21 years old individuals, Kitzbichler et al. [130] found in their NT control group an initially strong beta, theta, and delta-band connectivity involving frontal regions. This connectivity decreases later with maturation, presumably evolving toward more specific interconnections. This developmental change was not observed in ASD participants who initially started with a low frontal connectivity and stayed at this level.

## Discussion

### Summary of the main observations reported in the reviewed literature

Our systematic review of a large body of evidence suggests that ASD is characterized by a pattern of EEG/MEG functional connectivity that is in general more randomly organized, with abnormal connectivity often involving frontal or occipital regions–at least in adult samples–although abnormal connectivity patterns have been reported in almost every region. Abnormal lateralization of activity in resting-state and in specific tasks seems to be typical, with a very systematic report of elevated left-over-right EEG and MEG functional connectivity ratio in ASD. Both abnormal intra- and inter-hemispheric connectivity can be observed with a general trend toward underconnectivity, but with probable local or condition-specific (task, spectral band, brain region) overconnectivity. Underconnectivity can most reliably be observed in lower frequency bands (hypothesized to be preferentially involved in long-range integrative networks) whereas a stronger tendency for overconnectivity can be observed (see Fig 2) in high-frequency ranges (hypothesized to be generally associated with more localized processes). These findings appear to hold despite substantial variability across several methodological dimensions including recording characteristics and analytic approaches. More investigation on the relationship between over/underconnectivity and EEG/MEG frequencies is nevertheless needed to corroborate these conclusions. This study might further benefit from looking at potential modulation across regions of high-frequency activity by the phase of slower rhythms (i.e., between-region phase-amplitude coupling) [162], how these different frequencies relate to feed-forward and feed-back processes at play in information integration, and how these different observations may or may not be integrated in a theory-driven accounts, such as the predictive-coding framework [163–172].

### Heterogeneity in ASD

Despite these general patterns related to categorical diagnosis of ASD, several findings highlight the importance of considering variability in the ASD phenotype related to connectivity. For example, individuals with an Asperger’s diagnosis can be differentiated from those with other ASDs relatively accurately (92.3% in [98]) based on their patterns of connectivity. Although the authors did not provide detailed information on how groups compared with respect to ADOS scores, they used a large sample of ASD children (N = 430), which is likely to overlap with the Asperger’s sample (N = 26) regarding the severity of the condition. Similarly, ASD with Fragile X Syndrome or with a *de novo* chromosomal mutation causing agenesis of corpus callosum is another example of a subgroup of individuals with ASD that might be clearly defined from a clinical point of view. These persons reported a normal level of attention to details, with the preserved ability to appreciate the whole rather than a preoccupation with patterns or parts [132]. Thus, connectivity features shown to correlate with greater attention to details in ASD (e.g., eye-open resting-state alpha power and coherence in posterior regions [110]) are probably under-represented in this subgroup. Thus, it might become increasingly important to subgroup or control for the different conditions that are grouped under the ASD umbrella to better understand the impact of this heterogeneity on observed connectivity patterns and, hopefully, to reconcile some contradictions in the literature.

Further, abnormalities in EEG/MEG functional connectivity increase with increasing symptoms severity in autism. Measures of connectivity abnormalities reported in surveyed papers has been correlated with the presence of autistic traits in the NT population [89] and with the severity of autism symptoms in ASD samples: ADOS scores were shown to correlate with connectivity strength in gamma and beta bands [130], with theta-band coherence in left-anterior and right-posterior regions [129], with phase-amplitude coupling in the fusiform face area (for face-processing task) [125], and local functional connectivity based on phase-locking [126]; SRS scores correlated with regional average complexity and connectivity node strength [87]; visual reasoning and reading abilities correlated with lateralization of coherence in the parieto-temporal regions in gamma band; imagination measure on the parent-report adult AQ correlated with global connectivity in the right superior temporal gyrus [132].

### Limitations

The conclusions derived from our systematic review are limited by a few potential methodological confounders. These are discussed hereafter.

#### Interpretation of EEG/MEG functional connectivity

The hypothesis underlying every approach used to measure functional connectivity in EEG/MEG is that the estimated connectivity (i.e., the similarity between two or more time series, computed through correlation, coherence, or similar measures) is proportional to physiological connectivity between the brain areas generating these time series. There are nevertheless pitfalls in interpretation of connectivity measures due to our limited understanding of their underlying physiology, e.g., hidden sources, differential sensor sensitivity for different kind of synaptic activity, etc.

#### Statistical power

The low statistical power in the reviewed studies may be misleading. With a sample size typical of these studies (we take N = 15 per group for this example), the power of a two-tail two-sample t-test with a significance level at 0.05 is only 0.26 for a size-effect considered as medium (Cohen's d at 0.5). That is, three studies out of four will report negative results for medium-sized differences in brain connectivity. Controlling for multiple comparisons further reduces the power of these tests such that only very strong effects may be reliably detected in most studies. When applied to the Fig 2, this implies that potential counter-evidence regarding underconnectivity can only be provided by studies reporting overconnectivity (and vice-versa) for comparable frequency bands, and, importantly, not by studies reporting inconclusive results.

**Publication bias:** As mentioned previously, the context of individual studies may impact on obtained results. When results are grouped, these contexts are partly lost and this can produce a bias in the overall conclusions. That is, studies looking for specific information (e.g., gamma activity over long-distance connections) may report specific findings (e.g., under-connectivity) which would not be the main outcome if they had investigated a broader context (e.g., connectivity of gamma activity in general). This creates a potential publication bias where topic of high interest is generally more scrutinized, resulting in the publication of more positive findings.

#### Head and brain size

The significant bias in head and brain size between ASD and NT participants may result in biases impacting the distance between EEG sensors and the properties of electrical signal propagation from the cortex to the scalp. Differences in brain size (not head size) may also have some effect on the signal-to-noise ratio in MEG since the cortical surface is in average closer to the sensors for larger brains. This effect is expected to be more important in children since brain size differences between ASD and NT are larger at this age. Entering these factors as co-variates in statistical analyses is advisable to control their impact on group comparisons of brain connectivity. Although brain size might not be readily accessible in studies not including MRI scans, standard metrics for the head size are easily measured (e.g., standard head measuring procedures are defined in any 10/20 EEG electrode placement manual).

#### Volume conduction and assessment of short-range connectivity in EEG/MEG

We noted that, compared to long-range functional connectivity, studies of local connectivity are more scarce. This pattern might be related to intrinsic limitations of EEG and MEG recording modalities for evaluating short-range connectivity, particularly in coherence analyses performed at sensor level. To record an EEG/MEG potential, a large pool (around 50,000) of synchronously activated cells with parallel apical dendrites (i.e., pyramidal cells) spanning a cortical patch of 40–200 mm^2^ is required [173]. This has two implications. First, it means that simple power analysis could be used to assess local connectivity–with higher EEG/MEG power indicating larger local connectivity–if between-subject comparisons were not biased by various factors such as the conductivities of the skull and other tissues that can cause a general offset of power measurements. Although EEG/MEG power studies were out of the scope of the current review, it is relevant to note that a recent review of this literature has shown no consistent pattern of altered EEG/MEG power in ASD. It suggest that the literature is more supportive of an increased variability of EEG/MEG responses in ASD [174] (see, however, [175] for a recent study that challenges this theory).

Second, the localization on the scalp of such cortical sources is intrinsically limited by the spatial extent of the cell assembly necessary to generate a recordable potential. More importantly, this theoretical spatial resolution is affected by a smearing of the electrical activity as it travels from the cortical (or sub-cortical) sources to the sensors. For EEG, potentials are reaching sensors through omnidirectional volume conduction. For that reason, they spread over larger territories than the initial source area and create severe biases when estimating coherence between close sensors (< 10 cm) [176]. This phenomenon can induce false between-group differences if autism is correlated with differences in global electromagnetic properties of the head tissues, such as impedances of the different layer of tissues (scalp, skull, dura mater, etc.). Observed differences in extra-axial fluid in infants who develop ASD [177] is one such factor that may be confounding coherence results. The effect of field spread due to volume conduction is less severe in MEG, but is still present. At sensor-level, different approaches (e.g., phase lag index, imaginary part of coherency) have been devised to remove zero-lag activity between different sensors on account that such an activity can be associated with volume conduction. Such approaches, however, discard any potential physiological connectivity with zero-lag that can emerge in systems with feedback-loops such as neural networks [178]. Further, a zero-lag synchronization can be expected in neural oscillators generating large-scale EEG/MEG oscillations [179]. Such zero-lag functional connectivity can be observed experimentally for example in area 17 of the cat visual cortex where initial zero-lag interhemispheric synchronization of neuronal activity can be disrupted by sectioning the corpus callosum [180]. Further, the long-standing hypothesis that volume conduction propagates instantaneously for the frequency range of interest in EEG analysis–an hypothesis depending on the validity of the quasistatic approximation of the Maxwell equations for volume conduction–is challenged by recent experimental work showing propagation delays of volume conducted EEG waves [181].

One way to partly mitigate the problem of volume conduction would be to compute connectivity on EEG sources estimated using more accurate electromagnetic model of the propagation of electrical activity. Although the effect of field spread cannot be completely resolved by the current level of sophistication of source-reconstruction algorithms, such an approach should definitely be used to supplement sensor-level analyses as it reduces the impact of volume conduction and helps better link electrical activity with brain regions [182]. Further, using cortical sources would allow to compute the distance of connections along the cortical sheet, which would provide a much better estimated of distances for correlational analyses than the bird fly distance between sensors (i.e., two sensors above two nearby gyri may be close-by but connections must be significantly longer to follow the cortical sheet forming a sulcus between these two gyri).

#### Discriminant validity

Our review is limited to EEG/MEG functional connectivity in ASD, but future work should also help clarify cross-cutting issue common to multiple neurodevelopmental conditions. It remains to be established if the pattern of connectivity we observed in ASD is specific to the condition or alternatively reflects a more general pattern of changes common to a broad group of neurodevelopmental disorders [183].

## Conclusion and future directions

This review illustrates the large heterogeneity of both the methods and the results of studies investigating brain electrophysiological connectivity in ASD. Some of this variability might be reduced by further improving the methods adopted (e.g. using source reconstruction, better controlling for ASD phenotypes).

Research on electrophysiological functional connectivity in autism has been pioneered by EEG coherence analyses performed on small sensor grids, which provided crude connectivity assessment between very large brain regions (e.g., lobes). Currently, high-density grids are available in EEG and are included by default in every MEG system. Furthermore, algorithms for the estimation of cortical (and even sub-cortical [184]) sources from recorded activity have been developed and are now routinely used in a large body of studies presenting both time- and spatially-resolved brain activations and connectivity. Only one EEG study has benefited from this potential, whereas half of MEG studies did. Results from this latter subset of researches are generally more convincing, not only because source reconstruction helps in mitigating confounders such as volume conduction [182], but also because they associate observed activity with specific brain structures. Because they are non-invasive, cheap, and can be made widely available for clinical applications, small-grid sensor-level analyses have some potential for the development of biomarkers for diagnostic purposes and potentially to close a biofeedback loop in experimental therapeutic approaches. However, high-density grids combined with sensor-level and source-level analyses provide a much more fertile ground for building and testing hypotheses and theories on functional connectivity in autism.

Studies examining individual variability within ASD and across neurodevelopmental conditions remain very sparse and future studies need to pay more attention to mapping connectivity onto phenotypic differences. The use of functional connectivity features for diagnostic application is also relevant since high accuracy (85–95%) has been reported by many independent research teams [88,97,102,125,126,135]. However, how such biomarkers would perform in infants for an early diagnosis is still an open question and fraught with several pragmatic and ethical complications [185].

A clearer theoretical foundation is necessary to efficiently establishes the role of connectivity length with respect to over/under-connectivity using EEG/MEG functional connectivity. Strong theoretical grounding can help address outstanding questions in Table 1.

**Table 1:** Research questions needing clear answers to provide a solid foundation for linking connection length versus EEG/MEG functional connectivity in autism.

1. How could short and long-range connectivity be clearly defined based on unambiguous biological substrates, e.g., using anatomical concepts which can be directly measured/imaged such as cortical columns, gyri, cerebral lobes, etc.?
2. Are short and long-range connectivity distinct concepts (i.e., physiologically different) or is connectivity better captured as a dimension? Accordingly, should long versus short-range connectivity be assessed as a categorical problem (e.g., using ANOVA) or as a continuous one (e.g., using correlational analysis)?
3. What are the most appropriate methods to measure short and long-range connections in EEG/MEG? What confounds need to be more systematically controlled for (e.g., head circumference, brain volume)?
4. Should EEG/MEG power (a point measurement) or a connectivity metric (i.e., a two-point measurement) be used for the assessment of local activity?
5. Considering that volume conduction might have a non-zero-lag component and genuine connectivity is likely to have a zero-lag component, how should volume conduction be controlled for when measuring local connectivity?
6. How are frequency bands associated with EEG/MEG functional connection length and over/under-connectivity in autism given the current knowledge about the role of these different frequency bands in top-down/bottom-up integration/segregation and given the pathophysiology models accounting for autism symptomatology?

Further work is also needed to better understand the complex interactions between frequency bands, brain regions, physiology of EEG/MEG oscillators (e.g., roles of specific cell types, neurotransmitters, ion channels; see [186]), and how they relate to different cognitive processes. Specific task-/event-related protocol will need to be devised to disentangle these different dimensions in a principled way. This knowledge is instrumental in designing future studies that could link together ASD symptoms, brain processes, and connectivity abnormalities.

Finally, given the nonlinear evolution of brain properties (e.g., size, white matter integrity, connectivity, etc.), the developmental evolution of these properties strikes us as a very important area of investigation since these changes are generating confusion in interpretation of the current literature. Aside from helping to reconcile apparent contradictions in the literature, a better understanding of how developmental factors induce ASD-related brain changes early in development would provide invaluable insights on the pathogenesis of ASD. Performing such analyses in a multi-modal framework may also further our understanding of the dynamics of ASD-related abnormalities in brain connectivity and help resolve some of the apparent contradictions arising when comparing results across modalities.

## Supporting information

S1 File PRISMA 2009 checklist. (DOC)

S2 File Detailed synthesis per methodological dimensions. (DOCX)

S1 Table Included papers investigating EEG functional and/or effective connectivity in ASD. (DOCX)

S2 Table Included papers investigating MEG functional and/or effective connectivity in ASD. (DOCX)
