# Supplementary material for: Is functional brain connectivity atypical in autism? A systematic review of EEG and MEG studies
Source: PLoS One. 2017 May 3;12(5):e0175870. doi: 10.1371/journal.pone.0175870 (PMC5414938; doi:10.1371/journal.pone.0175870)
Supplement: S2 File — (DOCX) [file pone.0175870.s002.docx]

**Detailed synthesis per methodological dimensions**

**Connectivity metrics**

Many connectivity measures are available and associated with specific strengths and limitations (see S1 and S2 Tables in [187] for an overview). In EEG, most analyses used variants of coherency (n=22). Among these, two controlled for volume conduction, one by using only the imaginary part of coherency to cancel zero-lag coherence (i.e., to exclude the part of coherency which is generated by signal components that are in-phase) and another using reduced coherence. Of the remaining 20 studies, except for one study using phase coherence, all used standard magnitude squared amplitude of coherency, which can be impacted by volume conduction artifacts in uncontrolled ways. Aside from coherency-based measures, a few alternatives were also used such as versions of synchronization likelihood (n=5), correlations (n=2), phase lag index (n=3), mutual information (n=1), and transfer entropy (n=1). In some studies, functional connectivity was followed by graph analyses (n=6). All studies considered functional connectivity only, except for one study [188] which also reported on effective connectivity using transfer entropy.

MEG studies adopted a more varied set of connectivity measures including coherence (n=5), maximized imaginary coherence (n=1), phase lag index (n=4), phase-locking value (n=1), Granger causality (n=3), partial directed coherence (n=1), mutual information (n=1), as well as a stochastic model of brain dynamics based on a multivariate Ornstein-Uhlenbeck process to extract both the functional connectivity and the background neuronal noise (n=1). Here again, graph theory was often applied to functional connectivity matrices to derive network properties (n=6).

Although some of the approaches used in these different studies might be considered as relatively more advanced or have been specifically developed to overcome limitations of other methods (e.g., partial directed coherence versus standard coherence), it would be difficult to weight conclusions from these studies according to the measure they adopted since the choice of the “best” measure of connectivity is still largely a matter of opinion. It is generally recognized that verifying connectivity results with more than one metric is prudent since different approaches show increased sensitivity to different features of the signals (e.g., non-linearities) so that they can give different results, even when applied to the same dataset.

**Recording parameters**

**Recording reference (EEG)**

The choice of an appropriate EEG recording reference is especially important for coherence studies since spurious evaluation of connectivity can arise from using a common reference or an average reference computed with a too small number of channels [189-191]. Some coherence studies addressed the issue of volume conduction artifacts by computing the current source density (CSD; n=3). Other studies reference to the average amplitude (n=6) or to a common electrode on the ear-lobes (linked or not; n=8), mastoids (n=3), tip of the nose (n=1), and vertex (n=1). One study tested four different montages to verify the impact of the reference, whereas four studies did not disclose the reference they used in their EEG montage. More than half of the coherence studies are susceptible to spurious computation of connectivity due to the use of either a common reference or an average reference computed with a small number of channels. In general, if comparing groups of participants, the impact of this confounder could be expected to be limited since it applies equally to the different groups. However, in the present case, known differences in head and brain size between persons with ASD and NT participants might induce a systematic bias in volume conduction. Furthermore, even if difference in brain/head size was not an issue, spurious directions of effects (e.g., over versus underconnectivity) and localizations could not be completely ruled out.

**Recording grid (EEG)**

Slightly more than a third of the reviewed studies (n= 12) used high-density electrode grids (64 channels or more). The remaining studies used a rather crude sensor grid (e.g., 10-20 montages of 19 electrodes) which provides poor coverage of some regions (e.g., inferior regions of temporal lobes) and might therefore underestimate the group-differences in these regions.

**Computation of cortical sources**

Only one of the EEG studies reviewed used cortical source reconstruction, computed from a small recording grid (19 channels) and with an averaged MRI template (i.e., it does not consider individual differences in head and brain structure for estimating cortical sources). In contrast, more than half of the MEG studies (n=13) computed cortical sources based on individually recorded or averaged MRI, with further parcellation of the cortical sheet in functional or cytoarchitecturally distinct regions using atlas-guided segmentation. This subset of studies provides much richer information since they allow establishing relationships with underlying brain structures. Increased precision is expected from studies using realistic subject-specific reconstructions of the brain, which is the norm more than the exception for this body of MEG literature.

**Sampling rate**

All EEG studies used at least a 200 Hz sampling rate which is appropriate for investigating roughly up to 50 Hz (i.e., Nyquist theoretical limit is at 100 Hz but imperfect filtering generally warrants considering a much lower limit in practice). MEG studies generally used higher sampling frequency (500 Hz or more). This higher sampling frequency made reporting possible on functional connectivity in higher frequency bands, which provided support for overconnectivity in high-gamma band (see section 3.2), a missing piece of the puzzle in the EEG literature.

**Experimental paradigm**

Various paradigms and stimuli were used for recording EEG. These can be combined in relatively coarse categories: resting-state (n=9), event-related response (n=13), brain activity during sleep (n=5) or ongoing EEG during other activities (e.g., memory encoding or video watching) that have not been specifically timed-locked. In MEG, about half of the studies investigated resting-state activity and the other half were looking at event-related activity. Due to the enormous variability in stimulus characteristics, in subsequent analyses, results are reported as a function of sleep, resting-state, or event-related paradigms without analyzing in detail task-related differences.

**Sample characteristics**

**Age**

EEG studies included infants at risk for autism (6 to 17 months old) followed up longitudinally (n=3), young children with autism below 6 years or so (n=5), older children and adolescents below 16 years (n=12), and adults (n=6). Some studies (n=5) used a wide age range, grouping infants with children and/or adults.

In MEG, one research group used a custom child-size system to study young children between 3-6 years old (n=3). Other studies included older children and adolescents between 6-16 years (n=8) and adults 16 years and older (n=8). One study included participants with a very large age-range (4 to 74 years old). This variability in age (from infants to elderly) is expected to be an important source of variation in reported results.

**Sample size**

Sample sizes varied from less than 10 to more than 400 per group. Typically, groups included 10-20 participants. Importantly, these small sample sizes, coupled with the use of conventional statistical approaches (i.e., frequentist – as opposed to Bayesian – inference) preclude the use of negative findings as evidence for the null hypothesis. In this literature, false negatives are likely to be frequently observed.

**Phenotypic characteristics**

Given our interest in the broadest possible representation of the autism phenotype, aside from studies comparing ASD versus NT participants, we also included studies on variation in the neurotypical population as a function of autistic traits (n=1) and infants at-risk for ASD ascertained as having an older sibling with an ASD diagnosis (n=3). Most studies included only Asperger’s or high-functioning individuals but three studies also included low-functioning individuals, one included participants diagnosed with Tuberous Sclerosis Complex and ASD, and one studied ASD patients with agenesis of the corpus callosum. Potential impact of these phenotypic differences on connectivity findings is further discussed in section 4.1.

Taken together, studies reviewed are substantially heterogeneous along several methodological dimensions, which we take into consideration in addressing the main hypotheses.

**References**

187. Mohammad-Rezazadeh I, Frohlich J, Loo SK, Jeste SS. Brain connectivity in autism spectrum disorder. Curr Opin Neurol. England; 2016;29: 137–147. doi:10.1097/WCO.0000000000000301

188. Khadem A, Hossein-Zadeh G-A, Khorrami A. Long-Range Reduced Predictive Information Transfers of Autistic Youths in EEG Sensor-Space During Face Processing. Brain Topogr. 2016;29: 283–295. doi:10.1007/s10548-015-0452-4

189. Fein G, Raz J, Brown FF, Merrin EL. Common reference coherence data are confounded by power and phase effects. Electroencephalogr Clin Neurophysiol. 1988;69: 581–584. Available: http://www.ncbi.nlm.nih.gov/pubmed/2453336

190. Hu S, Stead M, Dai Q, Worrell GA. On the recording reference contribution to EEG correlation, phase synchrony, and coherence. IEEE Trans Syst Man Cybern B Cybern. 2010;40: 1294–1304. doi:10.1109/TSMCB.2009.2037237

191. Schiff SJ. Dangerous phase. Neuroinformatics. 2005;3: 315–318. doi:10.1385/NI:3:4:315
