# Supplementary material for: Is functional brain connectivity atypical in autism? A systematic review of EEG and MEG studies
Source: PLoS One. 2017 May 3;12(5):e0175870. doi: 10.1371/journal.pone.0175870 (PMC5414938; doi:10.1371/journal.pone.0175870)
Supplement: S1 Table — (DOCX) [file pone.0175870.s003.docx]

**Table S1. Included papers investigating EEG functional and/or effective connectivity in ASD.**

| **Paper** | **Connectivity metrics** | **Reference** | **Grid** | **Paradigm** | **Age** | **Sample size** | **IQ** | **Diagnosis** |
| --- | --- | --- | --- | --- | --- | --- | --- | --- |
| [88] | Fuzzy synchronization likelihood | N/A | 19 | Eyes-closed resting | 7-13 yo | 9 ASD; 9 NT | N/A | DSM-IV |
| [89] | Synchronization likelihood and graph measures | Average | 128 | Eyes-closed resting | 27±5 yo | 74 NT (37 F) | No IQ reported; educational level = 19.2 | SRS-A from someone who knew them well |
| [90] | Synchronization likelihood and graph measures | Average | 128 | Eyes-closed resting | 25±7 yo | 10 HF-ASD or AS; 10 NT (1 F/group) | **HF-ASD and AS:** Verbal: 88-116 (103.1 ± 9.5); exec: 74-131 (98.6 ± 22.8) | DSM-IV and ADOS-G (within the past 3 years) |
| [91] | Phase lag index and graph measures | CMS-DRL | 32 | Passive viewing of pictures of faces and cars | 3.4±1.0 yo | 19 NT (0 F); 12 ASD (2 F) | **NT:** 108.0 ± 12.4; ASD: 85.0 ± 17.2) [unspecified IQ metric] | DSM-IV diagnostic of autism (2), AS (1) or PDD (9) |
| [92] | Coherence, phase lab, Person's and partial correlation | N/A | 19 | Awake, REM, and SWS | 4±1 yo | 87 ASD (17% F); 21 noASD-DD (38% F); 29 NT (28% F) | No IQ reported; noASD-DD were recruited as IQ-matched comparisons | DSM-IV criteria for ASD, ADI-R (or a Toddler version), ADOS, and clinical judgment |
| [93] | Coherence | Average mastoids | 64 | Watching video of familiar/unfamiliar person reading a story | 10±1.6 yo | 19 HF-ASD (1 F); 13 NT (4 F) | IQ > 75 (Kaufman Brief Intelligence Test); no between-group IQ difference | ADOS |
| [94] | Wavelet coherence | Tip of the nose | 28 | Neutral face and chair matching task | 30±5 yo | 15 ASD; 15 NT | **NT:** verbal: 77-133 (114±16); performance: 93-134 (119±11), full-scale: 93-134 (119±14) : **ASD:** verbal:101-134 (119±11); performance: 93-132 (115±14), full-scale:98-136 (119±13)  [WASI] | DSM-IV; Autism Spectrum Quotient |
| [95] | Coherence; sLORETA source localization with an averaged MRI template | Linked-ears | 19 | Visual memory task involving line drawings. Experimental groups: Nei Gong (Chinese mind-body exercise) training (NGT), progressive muscle relaxation (PMR) training, or control | 5-17 yo | 66 ASD separated in 3 experimental groups:  NGT: 18 (1 F), PRM: 17 (2 F), Control: 13 (1 F) | **NGT:** 76.3±17.7, **PRM:** 86.5±17.5, **Control:** 85.9±29.3 [WISC, 4 ed., Chinese version] | ASD or PDD based on DSM-IV-TR criteria; ADI-R |
| [96] | Coherence | Linked-ears | 19 | Eyes-closed resting | 6-11 yo | 20 ASD; 20 NT [6 F/group] | Subject individually matched between group for IQ; ASD: 93±16.8; NT: 98±15.4  [full-scale] | ASD or autism based on DSM-IV |
| [97] | Coherence with PCA | CSD | 24 | Awake and alert state | 2-12 yo | 430 ASD (16% F); 554 NT (12% F) | N/A | ASD or PDD diagnostic based on DSM-IV  and/or ADOS |
| [98] | Coherence with PCA | CSD | 24 | Awake and alert state | 2-12 yo | 430 ASD (16% F); 554 NT (12% F) | N/A | ASD or PDD diagnostic based on DSM-IV  and/or ADOS |
| [99] | Imaginary part of coherency (IC) | CSD | 128 | Happy/fearful and familiar/unfamiliar face processing | 2-5 yo | 31 NT; 72 ASD | N/A | ADI and ADOS |
| [100] | Reduced coherence and phase synchrony | Average | 128 | Long latency flash ERP | 5.5-8.5 yo | 6 ASD (1 F); 8 NT (4 F) | **ASD:** 43–105 (67.8±27.2); **NT:** 106–134 (117.1±9.8) [Differential Abilities Scale - General Conceptual Ability] | ADI-R, ADOS and DSM-IV diagnostic for autism or ASD |
| [101] | Coherence | Cz, reformatted to average | 128 | Joint attention-eliciting video clip with congruent and incongruent gaze-target conditions | 16±2 yo | 16 ASD (2 F); 17 NT (6 F) | **ASD:** verbal: 99±14.6; performance: 106±13.2;  **NT:** verbal: 108±14.8; performance: 103±16.8;  [WISC-IV] | Previous ASD diagnostic; confirmed with ADOS and SCQ |
| [102] | phase synchronization [wavelet] and graph measures | N/A | 128 | Fearful, happy and neutral face processing | 6-13 yo | 12 ASD; 12 NT | N/A | N/A |
| [103] | Coherence | Average | 64 - 128 | Familiar/unfamiliar neutral faces | 6 and 12 mo (longitudinal) | **At 6 mo:** 27 HRA (15 F); 31 LRC (16 F);  **At 12 mo:** 39 HRA (19 F); 30 LRC (12 F) | **At 6 mo**: HRA: 81-122 (99±10); LRC: 77-115 (96±10); **At 12 mo:** HRA: 76-138 (102±15); LRC: 90-134 (110±12)  [MELC] | HRA has a sibling with an ASD, AS, or PDD diagnostic according to SCQ, SRS and/or ADOS |
| [104] | Transfer entropy and directed differential connectivity graph | Linked-mastoids | 32 | Event-related with upright and upside down faces | 9-17 yo | 12 ASD; 19 NT (all males) | IQ > 70 (Raven Progressive Matrices); significantly lower IQ in ASD group | DSM-IV, CARS, and ASDS |
| [105] | Mutual information (coarse-grained entropy information) | N/A | 21 | NREM sleep | 8±3 yo | 27 ASD; 20 deaf children with half of them having a mild form of dysphasia | **ASD:** 54% were consider having mild mental retardation; **NT:** mentally non-retarded | IDC-10 criteria for pervasive developmental disorders and CARS |
| [106, 107] | Coherence | Unilateral ear lobe | 14 | Rhythmic visual stimulation | 6-16 yo (10±3 yo) | 14 ASD; 19 NT (all males) | **ASD:** verbal: 91.2 ± 27.5; performance: 94.3 ± 20.4; total: 91.4 ± 22.8 | DSM-IV |
| [108] | Coherence | Linked-ears | 22 | REM sleep | 21±4 yo | 9 ASD; 13 NT; | **ASD:** performance: 107.5, global: 101.3; **NT:** performance: 113.3; global: 115.7 | ADOS-G and ADI-R |
| [109] | Coherence | Linked-ears | 19 | Fixation of a target and video watching with or without sound | 3-9 yo | 21 ASD (8 F) | IQ > 85 | DSM-V criteria |
| [110] | Coherence | Vertex | 128 | Eyes open and eyes closed resting state | 18-51 yo | 15 HF-ASD (3 F); 16 NT (4F) | **ASD:** 100.9±18.6; **NT:** 107.1±11.9  [Combined vocabulary and non-verbal object series/matrices subtests of the Stanford-Binet Intelligence Scale, 5th ed] | Autism, AS, ADF, or PDD diagnostic according DSM-IV |
| [111] | Correlation | Double banana, transverse, Hjorth Laplacian, and neck | 19 | Wake and NREM sleep | 4-8 yo | 27 ASD (2 F); 55 NT (26 F) | N/A | Diagnosed with ASD by a specialist in child neurology, child psychiatry, or developmental pediatrics. |
| [112] | Coherence | Average | 124 | Eyes-closed resting | 18-38 yo | 18 ASD; 18 NT (all males) | **ASD:** full-scale: 86-138 (107.3±14.0); verbal: 84-140 (108.3±16.0); performance: 83-128 (104.4±13.5);  **NT:** full-scale: 88-139 (106.1±13.6); verbal: 89-132 (106.7±12.1); performance: 78-136 (104.0±17.4) [WAIS] | Diagnostic interviews and DSM-IV criteria |
| [113] | Debiased weighted phase lag index | Vertex | 128 | Passive watching of “social” and “non-social” videos | 12-17 mo | 10 HRA-ASD (3 F); 18 HRA-noASD (15 F); 26 LRC (14 F) | **At 14 mo:** HRA-ASD: 86±15; HRA-noASD: 100±12; LRC: 104±17; **At 36 mo:** HRA-ASD: 100±27; HRA-noASD:110±18; LRC: 115±14 [MELC] | HRA-ASD vs. HRA-noASD diagnoses performed at 36 mo using DAWBA and parent-report SCQ |
| [114] | Coherence and graph measures | Average | 19 | Eyes-open resting state | 0.7-25.6 yo (mean: 6.9 yo) | 14 TSC-ASD (5 F); 29 TSC-noASD (12 F); 16 nsASD (4 F); 46 NT (27 F) | N/A | TSC confirmed genetically; ASD diagnosed with  DSM-IV and ADOS |
| [115] | Coherence | Average | 64 - 128 | Auditory stimuli in a double oddball paradigm | 6 and 12 mo (longitudinal) | 28 HRA (5 with ASD at 36 mo); 26 LRC | MSEL similar between groups. Evaluated 5 scales; See table 2 in [115] for full data. | Older sibling diagnosed with SCQ or ADOS-G |
| [116] | Coherence | Linked-ears | 19 | Visual encoding object recognition task | 5-14 yo (10 ± 2.2 yo) | 21 ASD (2 F); 21 NT (7 F) | **NT**: 106.0±14.59; **ASD**: 101.86±16.09 [TONI-III] | DSM-IV diagnostic of autism (16) or PDD (5) and CARS |
| [117] | Coherence | Linked-ears | 19 | Looking at a fixed car pictures | 8-17 yo (12 ± 2.6 yo) | 17 HF-ASD (3 F); 17 LF-ASD (2 F) | **HF-ASD**: 106±20; **LF-ASD**: 56±12 [WISC-III short form] | DSM-IV diagnostic of autism (20), AS (2) or PDD (12) and ADI-R |
| [118] | Phase coherence | Linked-mastoids | 10 | NREM sleep | 7.5–21.5 yo | 10 AS*; 14 NT | **NT**: 49.9 ± 6.5; **AS**: 51 ± 8.8 non-verbal IQ [mean Raven’s Progressive Matrices row score] | AS diagnosed with ICD-10, confirmed with ADOS |

ADI: Autism Diagnostic Interview; ADI-R: ADI – Revised; ADOS-G: Autistic Diagnostic Observation Schedule-Generic; AS: Asperger’s Syndrome; ASD: Autism Spectrum Disorder; ASDS: Asperger Syndrome Diagnostic Scale; CARS: Childhood autism rating scale; CMS-DRL: Common Mode Sense-Driven Right Leg; CSD: current source density; DAWBA: Development and Wellbeing Assessment; DSM-(IV/V): Diagnostic and Statistical Manual of Mental Disorders, (fourth edition/fifth edition); ERP: Event-related potential; HF-ASD: high-functioning ASD; HRA: high risk for autism – based on an older sibling being diagnosed with ASD; HRA-ASD: HRA at time of recording – diagnosed with ASD later; HRA-noASD: HRA at time of recording – diagnosed free of autism later; IC: imaginary part of coherency; IQ: Intelligence Quotient; LF-ASD: low-functioning ASD; LRC: Low-risk comparison – based on not having older siblings diagnosed with ASD; MSEL: Mullen Scales of Early Learning; MELC: Mullen Early Learning Composite; mo: month old; noASD-DD: non-ASD developmental disorder; nsASD: non-syndromic ASD; NREM: Non-REM sleep; NT: Neurotypical; PCA: Principal component analysis; PDD: Pervasive Developmental Disorder not otherwise specified; REM: rapid eye movement sleep; SCQ: Social Communication Questionnaire; SRS: Social Responsiveness Scale; SRS-A: SRS - adult version; SWS: slow wave sleep; TONI-III = Deviation quotient of the Test of Non-verbal Intelligence, 3rd edition; TSC: Tuberous Sclerosis Complex; WASI: Wechsler Abbreviated Scale of Intelligence; WAIS: Wechsler Adult Intelligence Scale; WISC-III: Wechsler Intelligence Scale for Children-Third Edition; yo: year old.

Note: When using the notation X ± Y, X represents the mean and Y the standard deviation. When using notation X-Y, X and Y are the borders of the range.

* The study includes 18 AS overall, but only data from 10 were included for the connectivity analysis.
