# Supplementary material for: Is functional brain connectivity atypical in autism? A systematic review of EEG and MEG studies
Source: PLoS One. 2017 May 3;12(5):e0175870. doi: 10.1371/journal.pone.0175870 (PMC5414938; doi:10.1371/journal.pone.0175870)
Supplement: S2 Table — (DOCX) [file pone.0175870.s004.docx]

**Table S2. Included papers investigating MEG functional and/or effective connectivity in ASD.**

| **Paper** | **Technique** | **Space** | **Paradigm** | **Age** | **Sample size** | **IQ** | **Diagnosis** |
| --- | --- | --- | --- | --- | --- | --- | --- |
| [119] | Phase lag index | Source (individual MRI; atlas based segmentation) | Dot counting forming animal-like or random patterns | 25±4 yo | 14 ASD; 14 NT (4 F/group) | **ASD:** 108 ± 14.2; **NT:** 120 ± 8.5 [WASI] | DSM-IV and ADOS |
| [120] | Frequency domain Granger causality | Source (averaged MRI model) | Picture naming | **ASD:** 28±13 yo; **NT:** 34±12 yo; **parents:** 38±6 yo | 12 ASD; 16 parents of ASD (not biologically related to the ASD participants); 35 NT | N/A | DSM-IV, ADI-R, and ADOS |
| [121] | Phase lag index | Source (individual MRI) | Set-shifting task | 6-14 yo | 16 ASD (3 F); 14 NT (2 F) | **ASD:** 107.0 ± 12.7; **NT:** 105.8 ± 15.6 [WASI] | N/A |
| [122, 123]* | Stochastic dynamical model of brain dynamics (multivariate Ornstein-Uhlenbeck process) | Sensor | Open-eyes resting | 6-16 (11±3) yo | 9 AS (0 F); 10 NT (4 F) | N/A | DSM-IV |
| [87] | Synchronization likelihood; multi-scale entropy | Sensor | Eyes-closed resting | 6-16 yo | 26 ASD; 22 NT | **ASD:** 67-138 (104 ± 17); **NT:** 85-132 (105 ± 13) [WISC-IV General Ability Index] | Interview, DSM-IV, CARS, and ADOS |
| [124] | Coherence | Sensor and source (individual MRI) | Saccade task with antisacade, prosacade and fixation conditions | 29±10 yo | 11 ASD; 11 NT (2 F/group) | **ASD:** 116 ±8; **NT:** 113±9 [estimated verbal IQ] | ADOS and ADI-R |
| [125] | Cross-frequency coupling | Source (individual MRI) | Presentation of images of houses and neutral, fearful and angry faces | 13-21 (17±2) yo | 17 ASD; 20 NT (all males) | **ASD:** verbal: 61-142 (112±21); nonverbal: 74-132 (112±16);  **NT:** verbal: 93-132 (115±10); nonverbal: 74-132 (112±18) [KBIT] | SCQ > 15 and ADOS |
| [126] | Non-parametric Granger causality; local functional connectivity index based on vibrotactile stimulation** | Source (individual MRI) | 25 Hz vibrotactile finger stimulation while watching a movie; resting state with a fixation target | 8-18 yo (mean: 11.6 yo) | 15 ASD; 20 NT (all males) | **ASD:** verbal: 83-141 (110.0±15.8); nonverbal: 73-144 (101.5±19.2);  **NT:** verbal: 80-141 (110.6±15.6); nonverbal: 77-140 (110.4±17.0) [KBIT] | SCQ > 15 and ADOS |
| [127, 128] | Coherence | Sensor | Watching a video of their choice | 3-7 yo | **First study:** 35 ASD; 35 NT (6 F/group); **Second study:** 26 ASD; 26 NT (4 F/group) | **First study:** ASD: mental processing: 92.7±21.0; achievement 93.9±22.2; NT: mental processing: 98.5±13.5; achievement 98.5±13.3;  **Second study:** ASD: mental processing: 95.0±19.2; NT: mental processing: 99.9±10.8 [K-ABC] | ADOS, DISCO, and DSM-IV |
| [129] | Coherence | Sensor | Watching a video of their choice | 3-7 yo | 50 ASD; 50 NT (11 F/group) | **ASD:** mental processing: 96.8±22.5; achievement 96.8±23.6; **NT:** mental processing: 98.4±14.1; achievement 100.1±12.8 [K-ABC] | ADOS, DISCO, and DSM-IV |
| [130] | Graph measures | Sensor and source (individual MRI) | Resting state with a fixation target | 6-21 yo | 15 ASD; 15 NT (all males) | N/A | SCQ > 15 and ADOS |
| [131] | Coherence | Source (average MRI model) | Passive viewing of direct and averted gaze | Mean: 17 yo | 10 ASD; 8 NT (3 F/group) | **ASD:** 79-144 (112.1±22.9); **NT:** 100-122 (116.9±7.1) [WASI full-scale] | PPD based on DSM-IV; ADI-R |
| [132] | Imaginary coherency | Source (individual MRI) | Eyes-closed resting | 4-74 yo | 47 child (45% ASD; 16 F); 20 adolescent (35% ASD; 7 F); 39 adults (18% ASD; 14 F); all with AgCC | IQ available in 75 subjects; 77% have IQ >= 70 | ASD: AQ; AgCC: expert consensus review of MRI scans |
| [133] | Phase lag index and graph measures | Source (individual MRI) with atlas-guided cortical segmentation | Perception of emotional faces (happy, angry, and neutral) | 14±1 yo | 22 ASD (4 F); 17 NT (3 F) | **ASD:** 94.7±14.6; **NT:** 112.5±11.1 [full-scale WASI] | expert clinical judgment, medical diagnostic  reports and  ADOS-G |
| [134] | Maximized imaginary coherence | Source (individual MRI) | Slit-viewing of line-drawings of objects, animal, and humans | 31±9 yo | 20 ASD; 20 NT (9 F/group) | **ASD:** IQ: 109.4±14.4 ; verbal IQ: 111.7±14.0; **NT:** IQ: 110.7±11.4 ; verbal IQ: 111.3±13.3 [WAIS] | AS or autism diagnosis (DSM-IV) |
| [135] | Granger causality and graph measures | Sensor | Resting state | 19±1 yo | 8 HF-ASD; 8 NT | Full-scale and verbal IQ > 85 | ADOS and ADI-R |
| [136] | Generalized synchronization (RIM), mutual information, partial directed coherence and graph measures | Sensor | Eyes-closed resting state | 17-21 yo | 8 HF-ASD; 8 NT (1 F/group) | **ASD:** full-scale 85-125 (104.6±15.8); verbal: 73-122 (101.6±16.8); performance: 83-126 (107.0±14.2); **NT:** full-scale 116-139 (126.6±8.4); verbal: 116-136 (122.7±8.0); performance: 116-132 (122.6±5.6) [WASI] | ADOS and ADI-R |
| [137] | Inter-regional PLV | Source (individual MRI) | 2-back task (key-press when detecting an image presented two trials earlier) | 7-13 yo | 17 ASD (4 F); 20 NT (7 F)*** | **ASD:** 109.94±13.92; **NT:** 115.95±10.97 | Previous ASD diagnosis; confirmation using ADOS-G and clinical judgment |
| [138] | Weighted phase lag index and graph analysis | Source (individual MRI) with atlas-guided cortical segmentation | Resting state with a fixation target | 12-15 (15±1) yo | 16 ASD (2 F); 15 NT (2 F) | Full-scale WASI IQ > 65 (according to rejection criteria) | Expert clinical judgment and/or ADOS-G |

ADI-R: Autism Diagnostic Interview – Revised; ADOS: Autistic Diagnostic Observation Schedule; AgCC: agenesis of the corpus callosum; AQ: Autism Spectrum Quotient; ASD: Autism Spectrum Disorder; CARS: Childhood Autism Rating Scale; dACC: dorsal anterior cingulate cortex; DISCO: Diagnostic Interview for Social and Communication Disorders; DSM-IV: Diagnostic and Statistical Manual of Mental Disorders, fourth edition; FEF: frontal eye field; FFA: fusiform face area; FG: fusiform gyrus; IFG: inferior frontal gyrus; IQ: Intelligence Quotient; K-ABC: Kaufman Assessment Battery for Children; KBIT: Kaufman Brief Intelligence Test; NT: Neurotypical; OCC: occipital lobes; PLV: Phase-locking value; RIM: robust interdependence measure; STG: superior temporal gyrus; yo: years old.

Note: When using the notation X ± Y, X represents the mean and Y the standard deviation. When using notation X-Y, X and Y are the borders of the range.

* The paper [123] has been included in combination with [122] although it would not fulfill inclusion criteria on its own (i.e., it presents an approach using a functional connectivity matrix without reporting results on functional connectivity but more in an information theoretic context). It has nevertheless been included jointly with the latter paper because it broadens their results on functional connectivity with assessment of information gain in autism, a closely related measurement.

** Based on the ratio of a higher frequency phase-locked component (50 Hz) to the sum of the phase-locked component at the stimulation frequency (25 Hz) plus the 50 Hz component. The authors consider this ratio to index local functional connectivity based on the work of [Langdon et al.](#_ENREF_86) (2011)****.

*** Rows of gender attribution in Table 1 of [137] appears to have been inverted, as indicated by mismatch between sample sizes. We reported here the number of female per group once this row inversion is corrected. We tried to verify this conclusion by sending an email to the corresponding author but we got no feedback.

**** Langdon AJ, Boonstra TW, Breakspear M. Multi-frequency phase locking in human somatosensory cortex. Prog Biophys Mol Biol. 2011;105: 58–66. doi:10.1016/j.pbiomolbio.2010.09.015
